# S100A8 and S100A9 in saliva, blood and gingival crevicular fluid for screening established periodontitis: a cross-sectional study

**DOI:** 10.1186/s12903-021-01749-z

**Published:** 2021-08-09

**Authors:** Hyun-Duck Kim, Sandeep Karna, YooJin Shin, Huong Vu, Hyun-Jae Cho, Sungtae Kim

**Affiliations:** 1 Department of Preventive and Social Dentistry, School of Dentistry, Seoul National University, 101, Daehak-ro, Jongno-gu, Seoul, 03080 Korea; 2 Dental Research Institute, Seoul National University, Seoul, Korea; 3 Department of Periodontology, Seoul National University Dental Hospital, Seoul, Korea

**Keywords:** Periodontitis, S100A8, S100A9, Saliva, Blood, Gingival crevicular fluid

## Abstract

**Background:**

Periodontitis is one of major oral diseases, which has no consensus on early screening tool. This study aimed to compare the association and screening ability of S100A8 and S100A9 in saliva, blood and gingival crevicular fluid (GCF) for periodontitis status.

**Methods:**

We recruited 149 community Korean adults, 50 no or initial periodontitis (NIPERIO) and 99 established periodontitis (PERIO). Using clinical attachment loss and a panoramic radiograph, stage II–IV of new classification of periodontitis proposed at 2018 was considered cases as PERIO. Enzyme linked immunosorbent assay kit was used to quantify S100A8 and S100A9. T-test, analysis of covariance, Mann–Whitney test and correlation analysis were applied to compare the relationship of S100A8 and S100A9 in saliva, blood, and GCF for periodontitis. Receiver operating characteristic curve was applied for screening ability.

**Results:**

Among S100A8 and S100A9 in saliva, blood and GCF, S100A8 in saliva was significantly higher in PERIO than in NIPERIO (*p* < 0.05). However, S100A8 and S100A9 in GCF were higher in NIPERIO (*p* < 0.05). The screening ability of salivary S100A8 was 75% for PERIO, while that of GCF S100A8 was 74% for NIPERIO. Salivary S100A8 was positively correlated to blood S100A8 (*p* < 0.05).

**Conclusion:**

Salivary S100A8 could be a potential diagnostic marker for established periodontitis and be useful for screening established periodontitis.

**Supplementary Information:**

The online version contains supplementary material available at 10.1186/s12903-021-01749-z.

## Background

Periodontitis, the major oral disease, is a polymicrobial infectious disease that is related with systemic inflammation, destroys supporting tissue around the tooth and ultimately leads to tooth loss [1, 2]. Systematic disease like diabetes, cardiovascular and stroke has been shown to be associated with periodontitis [3–5]. Polymicrobial biofilm interacts with periodontal tissue and the biofilm triggers the host response, which leads to elevate systemic inflammation through change in proteins, immunoglobulins and inflammatory mediators [6, 7]. Upon activation of inflammatory mediators, various degradation pathways are activated that causes secretion of destructive cellular molecules like protease, reactive oxygen species, chemokines and cytokines [8].

Early detection of periodontitis is necessary for public health in a preventive dimension, because it leads to tooth mobility, tooth loss, mastication deficiencies and digestive problems [9]. Periodontitis is conventionally identified by dentist, inspecting the tissues around the teeth and using radiograph to determine bone loose around the teeth. However, the clinical procedures of present diagnostic measures are time consuming and too delayed to be restored.

Biofluids like blood, saliva, urine, tears have been used as source of biomarkers for certain disease [10]. Scientists are focusing much attention on biofluids, compared to use of tissue because of several factors like ease of accessibility, low cost of obtainment, avoiding risk of biopsies, and availability of multiple sampling [11]. Saliva contains proteins, peptides, organic and inorganic salts, electrolytes from blood with additional contribution from mucosal transudates and gingival crevicular fluid (GCF) [12]. Therefore, saliva has been studied and used for diagnostic tools over last decade. Recently salivary biomarkers have been applied for cardiovascular disease, autoimmune diseases, diabetes, HIV, oral cancer, caries and periodontal diseases [13].

S100A8 and S100A9 are a subgroup of molecules within the broader family of S100 calcium-binding protein and it has ability to bind with zinc. These proteins are mostly expressed on neutrophils and monocytes or macrophages [14]. Previously it has been reported that increased concentration of S100A8/A9 in saliva and serum were associated with periodontitis patients [15, 16]. Similarly, GCF fluid containing S100A8 and S100A9 was associated with periodontitis [17]. Thus salivary S100A8 and S100A9 have been specific targets for researcher and practitioners who are interested to identify periodontitis using robust and cost-effective method [18]. One study reported that salivary calprotectin (S100A8/9) was compared with that in serum, which had only 100 participants [16]. There has been no comparative evidence on S100A8 and S100A9 among saliva, blood and GCF. Hence more evidence is needed to compare salivary S100A8 and S100A9 with those in blood and GCF from more sufficient number of participants. Thus, the hypothesis of this study was that S100A8 and S100A9 in saliva, blood and GCF show the difference in the association and screening ability of S100A8 and S100A9 in saliva, blood and GCF according to the periodontitis status.

Hence, the present study aimed to compare the association and screening ability of S100A8 and S100A9 in saliva, blood and GCF according to the periodontitis status.

## Methods

### Study design and ethical consideration

This cross-sectional study randomly selected participants from public advertisement. All of the participants voluntarily provided a written informed consent. The Institutional Review Board for Human Subjects of Seoul National University Dental Hospital reviewed and granted the ethical consideration for this study (CRI17009). This study was organized according to the checklist of items included in STROBE (cross-sectional studies) (Additional file 1).

### Sample size estimation

The results of pilot test using ten participants (each five cases and controls) showed that S100A8 in saliva [mean ± standard deviation (SD), ng/ml] was 8.0 ± 10.2 for established periodontitis (PERIO) patients versus 6.1 ± 8.3 for no or initial periodontitis (NIPERIO) participants. Under the condition of type I error of 0.05, type II error of 0.8 and ratio of 2 between PERIO and NIPERIO participants, the sample size of this study was estimated as total of 147 (98 PERIO and 49 NIPERIO). Sample size was estimated 18 (12 PERIO and 6 NIPERIO) for S100A8 in GCF (0.4 ± 0.4 for NIPERIO vs. 0.15 ± 0.3 for PERIO) and 27 (18 PERIO and 9 NIPERIO) for S100A8 in blood (600 ± 240 for PERIO vs. 490 ± 210 for NIPERIO). The estimated sample size for S100A9 was 141 (94 PERIO and 47 NIPERIO) in saliva (1.2 ± 1.9 for NIPERIO vs. 1.6 ± 2.1 for PERIO), 21 (14 PERIO and 7 NIPERIO) in GCF (0.2 ± 0.2 for NIPERIO vs. 0.08 ± 0.2 for PERIO) and 39 (26 PERIO and 13 NIPERIO) in Blood (159 ± 28 for NIPERIO vs. 170 ± 24 for PERIO). Finally, we decided the total samples size of this study was 147 (98 PERIO and 49 NIPERIO).

### Study participants

The total of 149 community Korean adults, 50 no or initial periodontitis and 99 established periodontitis were recruited. The inclusion criteria for participants of this study were five-fold. (1) Those who agreed to take periodontal examination including clinical attachment loss (CAL) and panoramic radiograph according to the new international periodontal classification guideline by American Academy of Periodontitis and European Federation of Periodontology [19], (2) Those who aged over 20 years, (3) Those who had no medication during previous three months, (4) Those who agreed to donate adequate sample of blood, GCF and saliva for analysis (5) Those who had no missing data used in the final analysis.

### Assessment of periodontitis

Trained dentists examined CAL and radiographic bone loss by using panoramic radiograph (Pax-Primo, Vatech Global, Seoul, Korea). CAL was calculated by adding up pocket depth and gingival recession using a UNC-15 probe in all of the natural teeth except 3rd molar. Periodontal status was categorised according to the guidelines of 2017 American Association of Periodontology—European Federation of Periodontology workshop in Periodontology [19]. Among our participants, only two participants were periodontally healthy and 48 participants were classified into Stage I (initial) periodontitis with CAL 1–2 mm and no history of extraction due to periodontitis. So, the participants with Stage II–IV periodontitis were considered as established periodontitis (PERIO) and the other participants with no or Stage I periodontitis were no or initial periodontitis (NIPERIO). Stage II–IV periodontitis is CAL ≥ 3 mm or extraction due to periodontitis or radiological bone loss > 15% of coronal third [19].

### Assessment of clinical periodontal parameters

Plaque index (PI), pocket depth (PD) and bleeding on probing (BOP) were considered as periodontal clinical parameters. PI was evaluated by Turesky modification of the Quigley–Hein Index [20]. PD was evaluated at six sites per tooth (mesio-, mid- and disto-buccal and lingual) using a UNC-15 probe and dichotomized according to PD** ≥ **4 mm. BOP was evaluated using the guideline in a previous study [21]. Finally, PI, PD and BOP were presented as PI, percentage of site with PD ≥ 4 mm and percentage of site with BOP positive.

### Saliva sampling

Information about the standard sampling protocol for saliva collection was provided to each participant: not to brush tooth, not to drink or eat one hour before sampling. In order to maintain consistency of samples, we collected unstimulated whole saliva using passive drooling method for 10 min in a 50 ml conical tube. We centrifuged the saliva by 2600×*g* for 15 min at 4 °C, aliquoted supernatants into 1 ml in sterilised 1.5 ml Eppendorf tube and stored the aliquoted saliva samples in – 80 °C deep-freezers for further analysis.

### GCF collection

Radiographic evaluation and periodontal probing using UNC-15 probe were applied to decide the deepest pocket among all teeth pockets. GCF samples were obtained from the deepest pocket. During GCF sampling, we tried to avoid blood and saliva contamination. After isolating the tooth with cotton rolls, three absorbent paper points (#25, Meta Biomed Inc., Chungbuk, Korea) were gently inserted in the same deepest pocket for 30 s. Paper points were immediately placed in a cryovial containing 1 ml of phosphate buffer saline in pH 7.4, which were centrifuged (2600×*g* for 15 min at 4 °C) and supernatants were aliquoted into 1 ml in sterilised 1.5 ml Eppendorf tube. The tubes with GCF samples were stored at stored at − 80 °C for further analysis.

### Blood collection

Blood of 4 ml was drawn by venepuncture by a trained medical technologist. The blood samples were centrifuged (2600×*g* for 15 min at 4 °C) and supernatants (plasma) were aliquoted into 1 ml in sterilised 1.5 ml Eppendorf tube. The tubes were then stored at − 80 °C for further analysis.

### Quantification of salivary S100A8 and S100A9

S100A8 and S100A9 protein concentrations were determined from saliva, blood and GCF using enzyme-linked immunosorbent assay (ELISA) kit (R&D systems, Minneapolis, MN, USA) with validation for cell culture supernatant and blood sample through manufacturer’s instruction. Standard curve was drawn using standard S100A8 and S100A9 supplied by the manufacturer. GCF and saliva samples were diluted on concentration dependent using reagent diluent provided by manufacture (1, 1/2, 1/4, 1/8, 1/16, 1/32) and diluted sample concertation for S100A8 and S100A9 were calculated from standard curve of S100A8 and S100A9. Similarly, blood samples were diluted on concentration dependent manner with reagent diluent (1/10, 1/20, 1/40, 1/80, 1/160, 1/320) and concentrations of S100A8 and S100A9 were estimated using standard curve. We decided the standard dilution rate that falls on the range of 500–1000 pg/ml on pilot study.

### Assessment of confounding variables

We considered sociodemographic factors such as sex and education, behavioural factors such as smoking and drinking, and systemic health information including obesity, diabetes, hypercholesterolemia and hypertension as confounders. Face to face interview, laboratory blood analysis and physical examination were applied to collect the data for confounders. We dichotomised following variables: education (until middle school vs. above high school), smoking (smoker encompassing past and current smoker versus non-smoker who has never smoked during the lifetime), drinking (alcohol drinker encompassing past and current drinker vs. non-drinker who has never drunk during the lifetime). We classified four systemic health components as follows: (1) obesity: body mass index (BMI) calculated as kg of body weight divided by square meter of height ≥ 25, (2) diabetes: high plasma glucose level (> 126 g/dl) or having anti-diabetic medication, (3) hypercholesterolemia: high plasma cholesterol level (> 240 mg/dl) or having anti-hypercholesterolemia medication, and (4) hypertension: systolic > 130 mmHg or diastolic > 85 mmHg or having anti-hypertensive medication. The blood pressure was measured by physicians in the sitting position using mercury manometer. We assayed biochemical variables using 12-h fasting blood samples drawn at recruitment.

### Statistical analysis

The distribution of characteristic variables by periodontitis status (NIPERIO vs. PERIO) were addressed using mean values with standard deviations (SD) for continuous variables, and frequencies and proportion for categorical variables. Chi-square test was applied for categorical variables. Kolmogorov–Smirnov (K–S) test was applied to evaluate the normal distribution for continuous variables. When variables were in normal distribution, parametric tests were applied, otherwise non-parametric tests were applied. T-test were performed to evaluate difference in continuous variables with normal distribution. Mann–Whitney (M–W) test were applied for continuous variables without normal distribution. The relationships between values in the blood, GCF and saliva were analysed with Spearman’s correlation test. Since number of participants were big (n = 149), Analysis of covariance (ANCOVA) was applied to estimate adjusted mean with standard error (SE) of S100A8 and S100A9 levels after controlling for age, sex, education, smoking, drinking, diabetes, hypercholesterolemia, hypertension and obesity. The receiver operating characteristic (ROC) curve was applied for estimating c-statistics (area under the curve: AUC) as screening ability of S100A8 and S100A9 for periodontitis status. Statistical significance was set at *p* value < 0.05. Data were analysed using Statistical Package for Social Sciences version 25 (SPSS inc, Chicago, Il, USA).

## Results

### Characteristic of participants

The participants of this study aged from 21 to 77 years. The PERIO participants were older, more males, more hypertensive and more obese than the NIPERIO participants (*p* < 0.05) (Table 1). The PERIO participants, compared to the NIPERIO participants, were higher educated, more diabetic, more hypercholesterolemia, more smokers and drinkers, which were not statistically significant (*p* > 0.05).

**Table 1:** Characteristics of the participants according to periodontitis status (n = 149)

Variable	NIPERIO (n = 50)	PERIO (n = 99)	*p* value
*Clinical parameter*^*a*^*, mean* ± *SD*			
Plaque index	**0.94 ± 0.72**	**1.26 ± 1.03**	**0.033**
Site with pocket depth ≥ 4 mm (%)	**0.24 ± 0.80**	**14.67 ± 16.02**	**< 0.001**
Bleeding site on probing (%)	**60.26 ± 30.22**	**71.07 ± 25.80**	**0.025**
Age^a^, mean ± SD	**42.1 ± 15.1**	**55.8 ± 11.6**	**< 0.001**
*Sex, n(%)*			**0.005**
Male	**15 (30.0)**	**54 (54.5)**	
Female	**35 (70.0)**	**45 (45.5)**	
*Education, n(%)*			0.054
Middle school	0 (0.00)	7 (7.1)	
High school or higher	50 (100.0)	92 (92.9)	
*Smoking, n(%)*^*b*^			0.262
No	48 (96.0)	90 (90.9)	
Yes	2 (4.0)	9 (9.1)	
*Drinking, n(%)*^*c*^			0.326
No	30 (60.0)	51 (51.5)	
Yes	20 (40.0)	48 (48.5)	
*Diabetic, n(%)*^*d*^			0.269
No	49 (98.0)	93 (93.9)	
Yes	1 (2.0)	6 (7.0)	
*Hypercholesterolemia*^*e*^*, n(%)*			0.102
No	49 (98.0)	90 (90.9)	
Yes	1 (2.0)	9 (9.1)	
*Hypertension*^*f*^*, n (%)*			**0.005**
No	**49 (98.0)**	**81 (81.8)**	
Yes	**1 (2.0)**	**18 (18.2)**	
*Obesity*^*g*^*, n (%)*			**0.031**
No	**40 (80.0)**	**62(62.6)**	
Yes	**10 (20.0)**	**37 (37.4)**	

Bold denotes statistical significance at *p* < 0.05

*PERIO* stage II–IV periodontitis (AAP-EFP, 2018), *NIPERIO* No or stage I periodontitis

*p* values: obtained by chi-square test for categorical variables and T-test for ^a^continuous variables. *SD* standard deviation

^b^Smoking: No = never smoked, Yes = past and current smoker

^c^Alcohol intake: No = never drunken, Yes = past and current drinker

^d^Diabetic: Yes = fasting plasma glucose > 126 or taking diabetes medication

^e^Hypercholesterolemia: Yes = plasma cholesterol > 240 or taking hypercholesterolemia medication

^f^Hypertension: Yes = systolic blood pressure ≥ 140 mmHg or diastolic blood pressure ≥ 90 mmHg or taking hypertension medication

^g^Obesity: Body mass index (body kg/height m^2^) ≥ 25

The PERIO patients, compare to the NIPERIO participants, showed significantly higher values in clinical periodontal parameters including PI, PD ≥ 4 mm and BOP (T-test, *p* < 0.05) (Table 1).

### S100A8 and S100A9 in saliva, blood, GCF by periodontitis

**Fig. 1:**
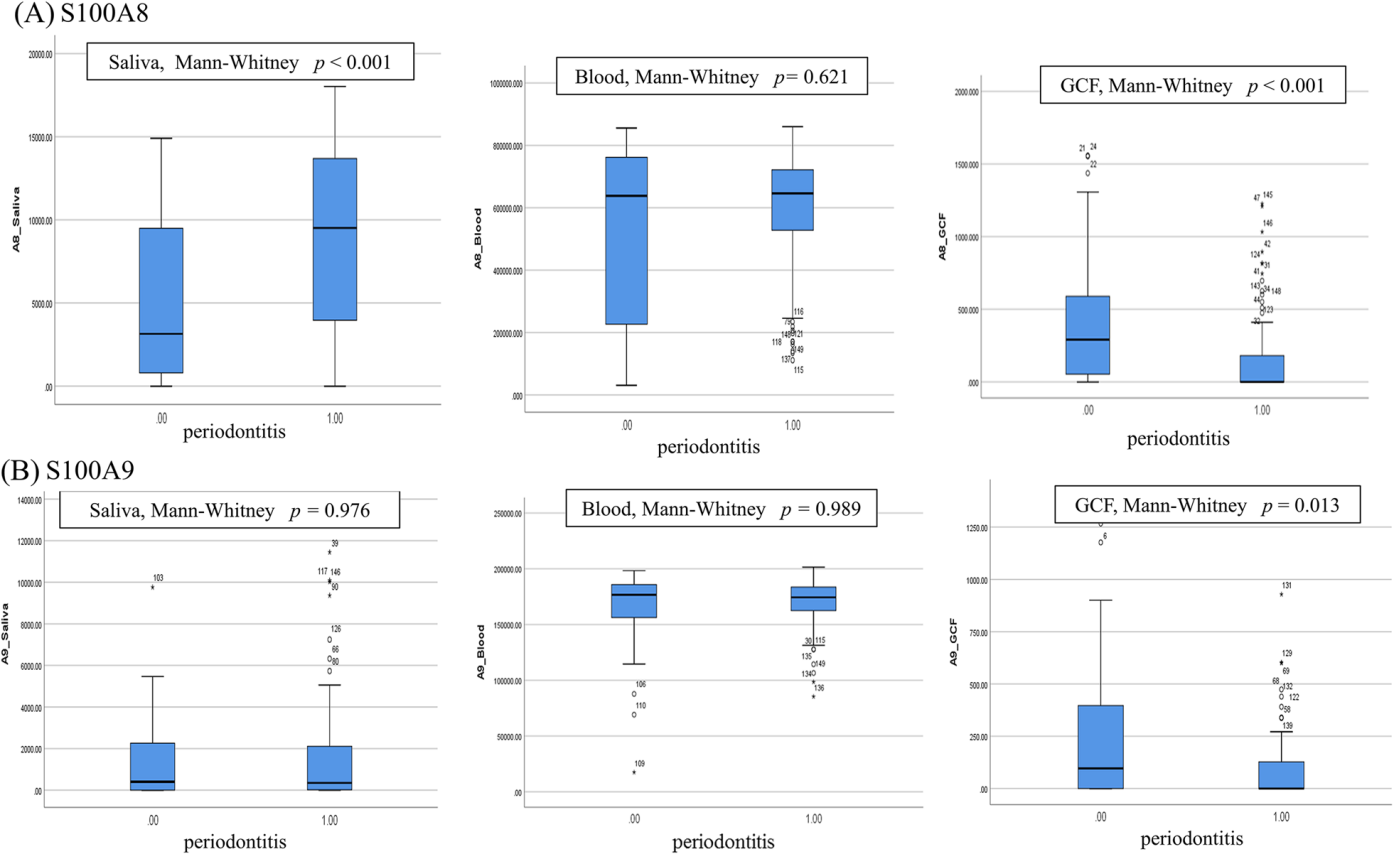
Distribution of S100A8 (pg/ml) and S100A9 (pg/ml) according to periodontitis status (0[n = 50]: no or stage I; 1[n = 99]: stage II–IV) (n = 149). **A** S100A8 in saliva (*p* < 0.001), blood (*p* = 0.621) and gingival crevicular fluid (GCF) (*p* < 0.001). **B** S100A9 in saliva (*p* = 0.976), blood (*p* = 0.989) and GCF (*p* = 0.013)

S100A8 and S100A9 in saliva, blood and GCF were not in normal distribution (K–S test, *p* < 0.05). The representative level of S100A8 in saliva was higher in PERIO participants than in NIPERIO participants (M–W test, *p* < 0.05) (Fig. 1). Although blood showed no difference in S100A8 according to periodontitis status, GCF showed was lower S100A8 in PERIO participants than in NIPERIO participants (M–W test, *p* < 0.05). However, S100A9 in GCF was lower in PERIO participants than in NIPERIO participants (M–W test, *p* < 0.05). S100A9 in saliva and blood showed no difference.

The adjusted value of S100A8 in saliva, after controlling for confounders, was also higher by 1.6 and 1.8 times in stage II and stage III–IV periodontitis participants than in NIPERIO participants (ANCOVA, *p* < 0.05) (Table 2). That of S100A9 in saliva showed no difference (ANCOVA, *p* > 0.05). In blood, the adjusted value of S100A8 and S100A9 were not significantly different according to periodontitis status (ANCOVA, *p* > 0.05). However, the adjusted values of S100A8 and S100A9 in GCF were higher by around 2.5 times in NIPERIO participants than in stage II and stage III–IV periodontitis participants (ANCOVA, *p* < 0.05).

**Table 2:** Differences in adjusted value (mean ± standard error) of S100A8 (pg/ml) and S100A9 (pg/ml) by periodontitis status (n = 149)

*Protein*	Periodontitis status			*p* value
Sample	No-Stage I (n = 50)	Stage II (n = 76)	Stage III–IV (n = 23)	
*S100A8 (pg/ml)*				
Saliva	**5348.53 ± 794.80**^**a**^	**8152.65 ± 607.91**^**b**^	**9512.13 ± 1132.64**^**b**^	**0.007**
GCF	**416.43 ± 56.02**^**a**^	**146.84 ± 42.85**^**b**^	**187.40 ± 79.84**^**b**^	**0.002**
Blood	497,523.22 ± 35,568.06	579,163.20 ± 27,204.67	648,641.67 ± 50,687.01	0.057
*S100A9 (pg/ml)*				
Saliva	1430.58 ± 393.97	1380.99 ± 301.33	1780.66 ± 561.44	0.820
GCF	**230.12 ± 36.41**^**a**^	**98.17 ± 27.85**^**b**^	**63.59 ± 51.89**^**b**^	**0.011**
Blood	162,405.26 ± 4146.13	172,167.34 ± 3171.22	166,097.21 ± 5908.53	0.189

Bold denotes statistical significance at *p* < 0.05

*p *values: obtained by ANCOVA for adjusted mean and standard error

Values were adjusted for age, sex, education, smoking, drinking, obesity, diabetes, hypercholesterolemia, and hypertension by ANCOVA in GLM. Superscript denotes same groups according to Bonferroni’s post hoc multiple comparison test

### Correlation between S100A8 and S100A9 in saliva, blood, GCF

**Fig. 2:**
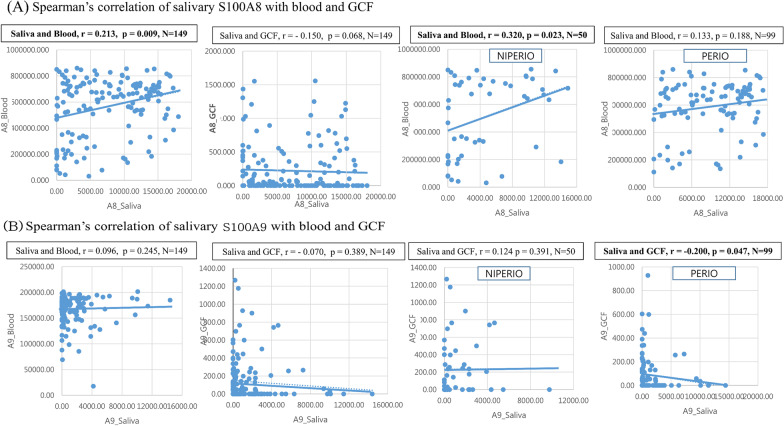
Correlation of salivary S100A8 (pg/ml) and S100A9 (pg/ml) with blood and gingival crevicular fluid (GCF) according to periodontitis status (NIPERIO[n = 50]: no or stage I; PERIO[n = 99]: stage II-IV) (n = 149). **A** Salivary S100A8. **B** Salivary S100A9. r: correlation coefficient

Scatter plot showed that S100A8 in saliva was positively correlated to that of blood (n = 149, r = 0.21, *p* < 0.05) (Fig. 2). This correlation increased by 50% in NIPERIO participants (n = 50, r = 0.32, *p* < 0.05). However, S100A9 in saliva was negatively correlated to that in GCF among PERIO patients (n = 99, r = −0.20, *p* < 0.05).

### Screening ability of S100A8 and S100A9 in saliva, blood, GCF for periodontitis

**Fig. 3:**
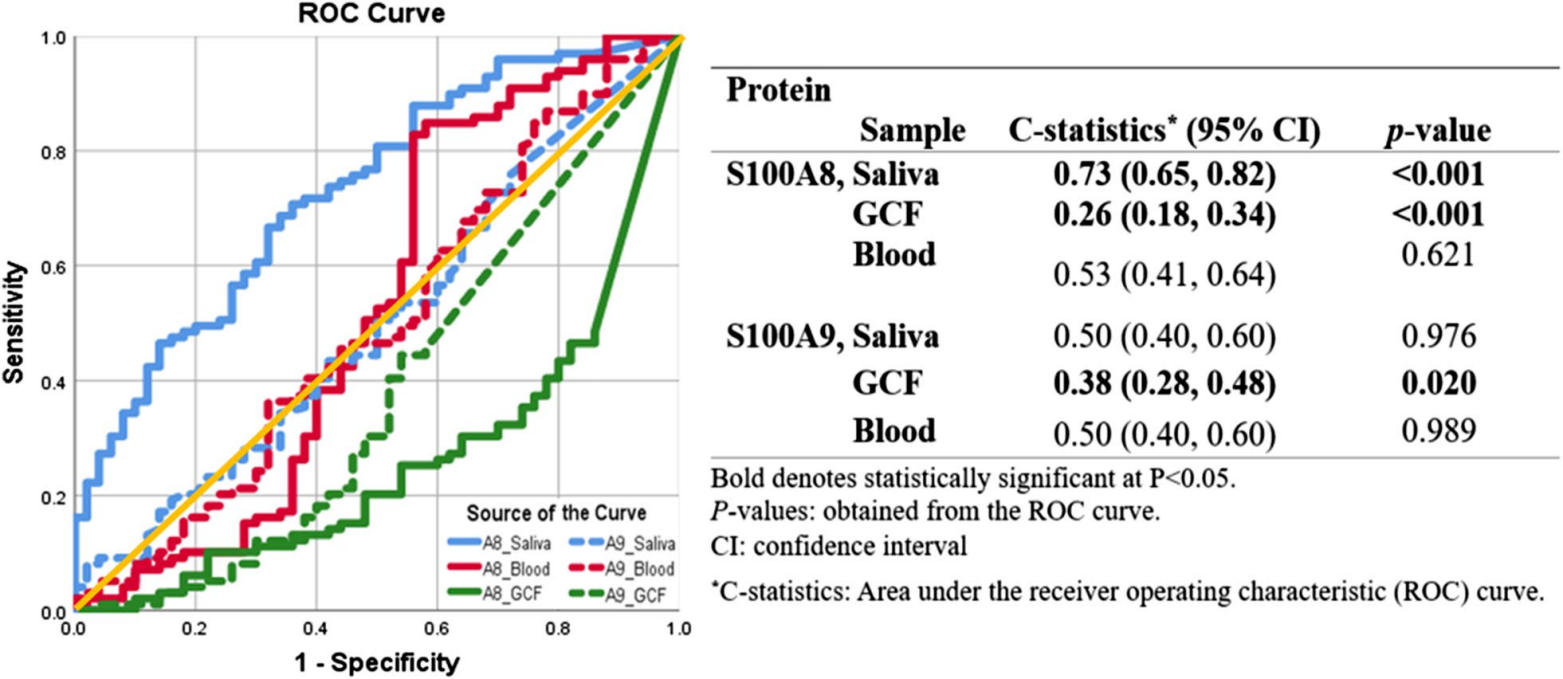
Receiver operating characteristic (ROC) curve for established periodontitis (stage II–IV) screening ability (c-statistics) of S100A8 and S100A9 in saliva, blood and gingival crevicular fluid (GCF) (n = 149)

ROC curve showed that salivary S100A8 had highest screening ability for PERIO among S100A8 and S100A9 in saliva, blood, and GCF (Fig. 3). The screening ability of salivary S100A8 for PERIO was c-statistics of 0.73 (*p* < 0.05), while that of salivary S100A9 was 0.50 (*p* > 0.05). S100A8 and S100A9 in GCF showed high screening ability of 0.74 and 0.62 for NIPERIO (c-statistics of 0.26 in S100A8 and 0.38 in S100A9 for PERIO, *p* < 0.05), respectively. S100A8 and S100A9 in blood showed non-significant c-statistics of 0.5 (*p* > 0.05).

## Discussion

Our data showed that salivary S100A8 had the most appropriate screening ability for periodontitis among S100A8 and S100A9 in saliva, blood and GCF. To the best of our knowledge, this is the first evidence that salivary S100A8 could be the best maker for screening established periodontitis after comparing among S100A8 and S100A9 in saliva, blood and GCF. This result was supported by the previous evidence that salivary S100A8/9, calprotectin, was a significant maker for periodontitis [16].

Comparing with the previous study, our study had some advantage. Firstly, this study compared S100A8 and S100 A9 levels among saliva, blood and GCF. Secondly, sufficient 149 number of participants were randomly recruited from the general population and there was no selection bias. Thus, our results could be generalized. Thirdly, age, sex, smoking, drinking, education, diabetic, hypercholesterolemia, hypertension and obesity were considered as confounders for the adjustment. Fourthly, physical and dental examination were performed by physicians and trained dentists using UNC-15 probes and a panoramic radiograph. Fifthly, periodontitis was classified according to the recent New international classification of periodontitis [19]. Finally, concentrations of S100A8 and S100A9 were quantify using ELISA kits at picogram level.

Our data showed that elevated levels of S100A8 in saliva were significantly associated with PERIO in Korean adults. A recent Korea study reported that salivary S100A8 levels were higher by 70% in periodontal disease than that of healthy participants [22]. S100A8 expression is up-regulated by oxidative stress, cytokine and growth factors [23] followed by activation of FcγRI and FcγRIV on macrophages through TLR-4 [24, 25], and enzymes from chondrocytes suggesting a role in pericellular matrix degradation [26]. Chinese and Swiss human studies [17, 27] reported positive results in GCF. An English study also showed that S100A8 in GCF was significantly higher in inflammatory gingival tissue than that of normal tissue [28]. However, our data showed that S100A8 in GCF was significantly higher in NIPERIO than in PERIO participants. Hence, more study on GCF S100A8 should be indicated to make certain the discrepancies of the results.

S100A9 involved in the regulation of inflammatory processes and immune response [29]. Calprotectin, S100A8/A9, is the marker for gingivitis and periodontitis [30, 31]. Since the main extracellular form of S100A8 and S100A9 is as a heterodimer (S100A8/A9 or calprotectin), the role of calprotectin should have been considered. Down regulation of S100A9 protein could indicate insufficient immunity stimulated by the infection [32]. This protein also promotes apoptosis and modulate the inflammatory response in periodontal ligament cells so its downregulation could suggest a suppression of inflammation [33, 34]. Antimicrobial activity of S100A9 also have been reported. The mechanism behind antimicrobial activity is the monomeric form of amyloid beta 1–42 that is negatively regulated by the innate immune system by downregulating the secretion of S100A9 [35]. However, our data showed that only GCF S100A9 level was significantly lower in PERIO than in NIPERIO. Recently, a Korean study reported that salivary S100A9 was also decreased in periodontitis patients compared to healthy participant [22]. However, our S100A9 data did not show significant difference in both saliva and blood. Thus, more studies are indicated to clarify these discrepancies.

Our data showed that S100A8 in saliva and blood was positively correlated each other. This link was higher in healthy adults compare to in periodontitis patients. These results showed the evidence that saliva represented local and systemic inflammation via GCF and blood, while blood represented only systemic inflammation. Hence, salivary S100A8 showed highest screening ability among S100A8 and S100A9 in saliva, GCF and blood. Contrary to previous studies [30, 31], salivary S100A9 in our data was negatively correlated to that of GCF, especially in periodontitis patients. Down regulation of S100A9 protein could indicate insufficient immunity [32] and could be prone to have periodontal inflammation. However, further studies are needed to elucidate the mechanism of these results. As to established periodontitis, salivary S100A8 could be the best consistent biological marker among S100A8 and S100A9 in saliva, GCF and blood.

Our data showed that the screening ability of S100A8 for established periodontitis was 0.73 of c-statistics, which was higher than the previous Korean study [22] with 0.6 of c-statistics and a bit lower than Austrian [16] calprotectin study with 0.86 of c-statistics. Since salivary S100A8 could be the best marker for periodontitis, a rapid test kit using salivary S100A8 could be effective on promoting periodontal health for general public. The next step of Salivary S100A8 research should be focused on whether salivary S100A8 could be the prognostic marker for periodontitis. The intervention of periodontitis using periodontal treatment will elucidate the role of S100A8 on periodontitis prognosis.

There are some limitations of this study. Firstly, samples were analysed using ELISA were stored more than one month. Long term storage of saliva might influence on the detection of salivary protein [36]. Secondly, elevated level of S100A8 and S100A9 observed in cancer and other inflammatory diseases. This could degrade diagnostic ability for periodontitis. Thirdly, the inclusion of a patient with stage I (initial) periodontitis in the control group can lead to misinterpretations and misclassification bias toward the null. Further studies should include only periodontally healthy participants in the control group for reducing this type of misclassification bias. Finally, Periapical radiograph is the ideal radiograph to assess radiographic periodontal bone loss (R-PBL). However, we selected panoramic radiograph instead of intraoral radiograph due to the time and efforts, because intraoral and panoramic R-PBL measurements have been demonstrated to be clinically coincident [37]. Notwithstanding these limitations, our data is appropriate to meet the objectives of this study.

## Conclusion

Overall, elevated level of salivary S100A8 protein concentration could be a valid marker for established periodontitis screening. Thus, S100A8 salivary kit will be useful for screening established periodontitis. Further prospective studies including periodontal treatment will be indicated for elucidating the prognostic effect of salivary S100A8 for the promotion of periodontal health.

## Supplementary Information

**Additional file 1.** STROBE Statement—Checklist of items that should be included in reports of observational studies.

## Data Availability

The datasets used and/or analysed during the current study are available from the corresponding author on reasonable request.

## Abbreviations

ANCOVA: Analysis of covariance
AUC: Area under the curve
BOP: Bleeding on probing
CAL: Clinical attachment loss
ELISA: Enzyme-linked immunosorbent assay
GCF: Gingival crevicular fluid
K–S: Kolmogorov–Smirnov
M–W: Mann–Whitney
NIPERIO: No or initial periodontitis
PD: Pocket depth
PERIO: Established periodontitis
PI: Plaque index
ROC: Receiver operating characteristic
R-PBL: Radiographic periodontal bone loss
SD: Standard deviation
SE: Standard error
