# Supplementary material for: S100A8 and S100A9 in saliva, blood and gingival crevicular fluid for screening established periodontitis: a cross-sectional study
Source: BMC Oral Health. 2021 Aug 9;21:388. doi: 10.1186/s12903-021-01749-z (PMC8351418; doi:10.1186/s12903-021-01749-z)
Supplement: Supplementary file 1 — Additional file 1. STROBE Statement—Checklist of items that should be included in reports of observational studies. [file 12903_2021_1749_MOESM1_ESM.docx]

STROBE Statement—Checklist of items that should be included in reports of observational studies

|  | Item No | Recommendation | Response |
| --- | --- | --- | --- |
| **Title and abstract** | 1 | (*a*) Indicate the study’s design with a commonly used term in the title or the abstract | Yes. In the title and the abstract. |
|  |  | (*b*) Provide in the abstract an informative and balanced summary of what was done and what was found | Yes. In the abstract |
| Introduction | | |  |
| Background/rationale | 2 | Explain the scientific background and rationale for the investigation being reported | Yes. In introduction.  Page 3- 4 |
| Objectives | 3 | State specific objectives, including any pre-specified hypotheses | Yes. In introduction.  Page 4 |
| Methods | | |  |
| Study design | 4 | Present key elements of study design early in the paper | Yes. In the methods  Page 4-5 |
| Setting | 5 | Describe the setting, locations, and relevant dates, including periods of recruitment, exposure, follow-up, and data collection | Yes. In the methods  Page 4-5 |
| Participants 6 | | (*a*) *Cohort study*—Give the eligibility criteria, and the sources and methods of selection of participants. Describe methods of follow-up  *Case-control study*—Give the eligibility criteria, and the sources and methods of case ascertainment and control selection. Give the rationale for the choice of cases and controls  *Cross-sectional study*—Give the eligibility criteria, and the sources and methods of selection of participants | Yes. In the methods.  Page 4-5 |
|  |  | (*b*) *Cohort study*—For matched studies, give matching criteria and number of exposed and unexposed  *Case-control study*—For matched studies, give matching criteria and the number of controls per case | N/A |
| Variables | 7 | Clearly define all outcomes, exposures, predictors, potential confounders, and effect modifiers. Give diagnostic criteria, if applicable | Yes. In the methods.  Page 5-7 |
| Data sources/ measurement | 8* | For each variable of interest, give sources of data and details of methods of assessment (measurement). Describe comparability of assessment methods if there is more than one group | Yes. In the methods.  Page 5-8 |
| Bias | 9 | Describe any efforts to address potential sources of bias | Yes. In the discussion.  Page 11 |
| Study size | 10 | Explain how the study size was arrived at | Yes. In the methods.  Page 4 |
| Quantitative variables | 11 | Explain how quantitative variables were handled in the analyses. If applicable, describe which groupings were chosen and why | Yes. In the methods.  Page 6-7 |
| Statistical methods | 12 | (*a*) Describe all statistical methods, including those used to control for confounding | Yes. In the methods.  Page 7-8 |
|  |  | (*b*) Describe any methods used to examine subgroups and interactions | N/A |
|  |  | (*c*) Explain how missing data were addressed | Yes. In the methods  Page 5 |
|  |  | (*d*) *Cohort study*—If applicable, explain how loss to follow-up was addressed  *Case-control study*—If applicable, explain how matching of cases and controls was addressed  *Cross-sectional study*—If applicable, describe analytical methods taking account of sampling strategy | Yes, In the methods  Page 4-5 |
|  |  | (*e*) Describe any sensitivity analyses | N/A |
| Results | | |  |
| Participants | 13* | (a) Report numbers of individuals at each stage of study—eg numbers potentially eligible, examined for eligibility, confirmed eligible, included in the study, completing follow-up, and analysed | Yes. In the methods.  Page 4-5 |
|  |  | (b) Give reasons for non-participation at each stage | Page 5 |
|  |  | (c) Consider use of a flow diagram | N/A. |
| Descriptive data 14* | | (a) Give characteristics of study participants (eg demographic, clinical, social) and information on exposures and potential confounders | Yes. In the results  Page 8, Table 1 |
|  |  | (b) Indicate number of participants with missing data for each variable of interest | N/A. |
|  |  | (c) *Cohort study*—Summarise follow-up time (eg, average and total amount) | N/A. |
| Outcome data | 15* | Report numbers of outcome events or summary measures | Yes. In the results  Page 8-9, Table 2 |
| Main results | 16 | (*a*) Give unadjusted estimates and, if applicable, confounder-adjusted estimates and their precision (eg, 95% confidence interval). Make clear which confounders were adjusted for and why they were included | Yes. In the results  Page 8-9, Table 2 |
|  |  | (*b*) Report category boundaries when continuous variables were categorized | Yes. In methods  Page 7 |
|  |  | (*c*) If relevant, consider translating estimates of relative risk into absolute risk for a meaningful time period | N/A. |
| Other analyses | 17 | Report other analyses done—eg analyses of subgroups and interactions, and sensitivity analyses | N/A |
| Discussion | | |  |
| Key results | 18 | Summarise key results with reference to study objectives | Yes. In the discussion.  Page 9-10 |
| Limitations | 19 | Discuss limitations of the study, taking into account sources of potential bias or imprecision. Discuss both direction and magnitude of any potential bias | Yes. In the discussion.  Page 11-12 |
| Interpretation | 20 | Give a cautious overall interpretation of results considering objectives, limitations, multiplicity of analyses, results from similar studies, and other relevant evidence | Yes. In the discussion.  Page 11-12 |
| Generalisability | 21 | Discuss the generalisability (external validity) of the study results | Yes. In the discussion.  Page 9-10 |
| Other information | | |  |
| Funding | 22 | Give the source of funding and the role of the funders for the present study and, if applicable, for the original study on which the present article is based | Yes. In acknowledgement  Page 13 |

* Give information separately for cases and controls in case-control studies and, if applicable, for exposed and unexposed groups in cohort and cross-sectional studies.

**Note:** An Explanation and Elaboration article discusses each checklist item and gives methodological background and published examples of transparent reporting. The STROBE checklist is best used in conjunction with this article (freely available on the Web sites of PLoS Medicine at http://www.plosmedicine.org/, Annals of Internal Medicine at http://www.annals.org/, and Epidemiology at http://www.epidem.com/). Information on the STROBE Initiative is available at www.strobe-statement.org.
